# Metformin’s effects on varicocele, erectile dysfunction, infertility and prostate-related diseases: A retrospective cohort study

**DOI:** 10.3389/fphar.2022.799290

**Published:** 2022-07-22

**Authors:** Chin-Hsiao Tseng

**Affiliations:** ^1^ Department of Internal Medicine, National Taiwan University College of Medicine, Taipei, Taiwan; ^2^ Division of Endocrinology and Metabolism, Department of Internal Medicine, National Taiwan University Hospital, Taipei, Taiwan; ^3^ Division of Environmental Health and Occupational Medicine of the National Health Research Institutes, Zhunan, Taiwan

**Keywords:** metformin, varicocele, erectile dysfunction, infertility, benign prostate hyperplasia, prostate cancer

## Abstract

**Objectives:** To investigate the risk of varicocele, erectile dysfunction (ED), infertility, prostatitis, benign prostate hyperplasia (BPH) and prostate cancer associated with metformin use.

**Materials and methods:** A total of 261,838 males, mean age 52.39 years (SD: 11.39), with a new-onset type 2 diabetes mellitus in 1999–2009 were identified from Taiwan’s National Health Insurance. Among them, 175,171 were metformin initiators [metformin (+)] and 86,667 were non-metformin initiators [metformin (−)] in the initial 12-month prescriptions of antidiabetic drugs. Follow-up started after the initial 12-month prescriptions. Outcomes were followed up until 31 December 2011. Intention-to-treat (ITT) and per-protocol (PP) hazard ratios comparing metformin (+) to metformin (−) were estimated by Cox regression incorporated with the inverse probability of treatment-weighting using propensity scores.

**Results:** The median follow-up time ranged 5.55–6.82 years in metformin (−) and 4.36–5.17 years in metformin (+) for different outcomes in ITT analyses. The respective median follow-up time in PP analyses ranged 2.20–2.61 years in metformin (−) and ranged 3.99–4.65 years in metformin (+). In the ITT analyses, for metformin (−), the incidence rates (per 100,000 person-years) of varicocele, ED, infertility, prostatitis, BPH and prostate cancer were 26.42, 455.89, 22.82, 590.23, 4226.19, and 141.69, respectively; and the respective incidence rates for metformin (+) were 25.65, 488.10, 32.60, 510.30, 3685.66, and 116.57. The hazard ratios (95% confidence intervals) comparing metformin (+) to metformin (−) in the ITT analyses were 0.960 (0.784–1.174) for varicocele, 1.077 (1.026–1.130) for ED, 1.368 (1.116–1.676) for infertility, 0.887 (0.849–0.927) for prostatitis, 0.883 (0.868–0.899) for BPH and 0.878 (0.802–0.961) for prostate cancer. The hazard ratios for the respective outcomes in the PP analyses were 0.845 (0.662–1.078), 1.350 (1.264–1.441), 1.396 (1.078–1.808), 0.800 (0.756–0.846), 0.875 (0.855–0.895), and 0.613 (0.548–0.686).

**Conclusion:** Metformin use in patients with type 2 diabetes mellitus is associated with a neutral effect on varicocele, a higher risk of sexual dysfunction (ED and infertility) and a reduced risk of prostate-related health (prostatitis, BPH and prostate cancer).

## 1 Introduction

Sexual function, infertility and prostate diseases are major clinical issues related to men’s urological health. These urological problems may share common pathological process with type 2 diabetes mellitus (T2DM) including endothelial dysfunction, insulin resistance and inflammation (Hackett, 2020). Furthermore, the comorbidities (e.g., obesity, hypertension and dyslipidemia), chronic vascular complications (heart disease, stroke, peripheral arterial disease, kidney disease and neuropathy), metabolic control status and the medications used for the treatment of related clinical problems in patients with T2DM may also predispose to the development of these urological problems (Leisegang et al., 2021; Sooriyamoorthy and Leslie, 2021).

Metformin is currently the most prescribed antidiabetic drug, being used in more than 150 million diabetes patients (He, 2020). Because metformin can exert multiple pleiotropic benefits beyond glycemic control, it is now recommended as the first-line treatment of T2DM. These benefits include reduction of insulin resistance, improvement of endothelial dysfunction, modulation of immune function, anti-inflammation, anti-atherosclerosis, anti-aging, anti-microbia, and anti-cancer (Nath et al., 2020).

After oral intake and gastrointestinal absorption, metformin distributes to various tissues including sexual organs of female ovaries and uterus and male testes and prostate (Tseng, 2022). In female patients with polycystic ovary syndrome and infertility, metformin is now recommended as an adjunct to ovulation (Faure et al., 2018). According to recent studies, metformin may also have some benefits in men’s health with regards to sexual function, infertility and prostate-related health (Tseng, 2022). However, studies for some outcomes are still rare [e.g., erectile dysfunction (ED) and infertility] or lacking (e.g., varicocele and prostatitis) (Tseng, 2022). The results for some outcomes that have been more researched, e.g., benign prostate hyperplasia (BPH) and prostate cancer, remain inconclusive (Tseng, 2022). Therefore, current evidence is not sufficient to recommend the use of metformin in related urological problems in men. Furthermore, most previous studies investigated a specific urological problem without taking into account the competing risk of death and none of them has ever investigated simultaneously the occurrence of all related urological outcomes in a single study.

By using the nationwide longitudinal database of Taiwan’s National Health Insurance (NHI), the present study was aimed at investigating the effects of metformin on men’s urological health including varicocele, ED, infertility, prostatitis, BPH and prostate cancer at the same time in patients with T2DM. Because these multiple outcomes may have different pathogenesis and the effects of metformin on different outcomes may require different periods of time of exposure, each outcome was investigated separately and not as a composite outcome. We investigated the different outcomes simultaneously in a single study so that the potential differences in impacts of metformin could be highlighted.

## 2 Materials and methods

### 2.1 National health insurance in Taiwan

This is a retrospective cohort study using the reimbursement database of the NHI. The NHI, implemented since 1 March 1995, is a nationwide healthcare system that covers >99% of Taiwan’s population. All hospitals and >93% of outpatient clinics in Taiwan have contracts with the Bureau of the NHI. For reimbursement purposes, the medical settings have to submit computerized records of all disease diagnoses, prescribed medications and performed procedures. The database can be used for research purposes if approval is granted after ethics review. This study was granted an approval number 99274 by the Research Ethics Committee of the National Health Research Institutes (NHRI) with consent waiver. All methods were carried out in accordance with relevant guidelines and regulations. Personal information in the database has been de-identified and informed consent is not required according to local guidelines and regulations. The database has been described in more detail previously (Tseng, 2015a).

### 2.2 Disease codes

The International Classification of Diseases, Ninth Revision, Clinical Modification (ICD-9-CM) is the disease coding system used throughout the study period. The respective ICD-9-CM codes for the diseases mentioned in the study are shown in Supplementary Table S1. Some previous studies have assessed the accuracy of the ICD-9-CM codes labelled in the NHI database for various disease diagnoses (Chang, 2004; Hsieh et al., 2019). The codes of 250. XX used for the diagnosis of diabetes mellitus have a sensitivity of 90.9% and a positive predictive value of 90.2% (Hsieh et al., 2019). In another study, moderate to substantial Kappa values ranging from 0.55 to 0.86 were noted for the agreements between claim data and medical records (Chang, 2004).

### 2.3 Outcomes investigated and groups compared

The risk of each urological outcome (i.e., varicocele, ED, infertility, prostatitis, BPH and prostate cancer, respectively) was compared between metformin initiators [denoted by metformin (+)] and non-metformin initiators [denoted by metformin (−)]. The antidiabetic drugs used by the patients during the first 12 months of drug prescription were used to classify the patients into metformin (+) and metformin (−). Metformin (+) referred to patients who had received metformin treatment during the first 12 months of prescription of antidiabetic drugs and metformin (−) referred to those who received no metformin treatment during the first 12 months of prescription of antidiabetic drugs (Tseng, 2019).

### 2.4 Enrollment of research subjects

#### 2.4.1 Inclusion criteria

For academic research, the NHRI created a cohort of 120,000 newly diagnosed diabetic patients randomly selected from the NHI database of the whole nation in each calendar year since 1999 [for more detail, please refer to (Tseng, 2015a)]. The patients should not have a diagnosis of diabetes mellitus in the previous years and the definition of diabetes mellitus was based on one of the following two criteria: 1) diagnosis of diabetes mellitus during an admission to the hospital or having been prescribed antidiabetic drugs during hospitalization; or 2) in an outpatient setting within 1 year, the patients have been diagnosed as having diabetes mellitus for two or more times, or diagnosed once as having diabetes mellitus plus being prescribed antidiabetic drugs once (Tseng, 2015a).

The inclusion criteria were the cohorts of newly diagnosed diabetes patients created randomly from the whole nation from 1999 to 2009 by the NHRI (Tseng, 2019). Therefore, a total of 1,320,000 patients (120,000 patients X 11 years) were first included.

The period from 1999 to 2009 was used to enroll research subjects based on the following reasons: First, a consensus statement of the American Diabetes Association and the European Association for the Study of Diabetes first suggested that metformin might be used as the initial therapy for the management of T2DM because of its low cost in 2008 (Nathan et al., 2008). However, metformin was not officially recommended as the initial drug monotherapy for T2DM until after 2012 because of its potential cardiovascular benefits (Inzucchi et al., 2012). Therefore, the prescription of antidiabetic drugs to patients with new-onset T2DM diagnosed after 2010 might have been affected by the new recommendations, leading to the enrollment of patients with more advanced T2DM in the metformin (−) group and thus would raise severe confounding and selection bias in the patients being compared.

The longitudinal reimbursement records from 1995 to 2011 of these patients were retrieved for analyses in the study. The database of the first 4 years (i.e., 1995–1998) after the implementation of the NHI was not used for the enrollment of study subjects in consideration that the database during the initial phase of NHI might not be accurate and complete. However, the database of these 4 years was used to ascertain a new diagnosis of diabetes mellitus and a new user of antidiabetic drugs starting after 1999. To assure that the patients were new cases of diabetes mellitus and the antidiabetic drugs were first prescribed during the enrollment period from 1999 to 2009, we looked back in the available NHI database for verification up to the first year since the implementation of NHI in Taiwan in 1 March 1995.

#### 2.4.2 Exclusion criteria

In consideration that some patients might have been given insulin or other antidiabetic drugs during an admission for some medical conditions but they might not be real cases of diabetes and that some diabetes patients might have been treated non-pharmacologically, we conducted the first step exclusion to identify patients with a more stringent diagnosis of diabetes mellitus. The following patients were excluded at this first step exclusion: 1) patients who had been prescribed antidiabetic drugs during hospitalization but had not been followed up at the outpatient clinics with a diagnosis of diabetes mellitus; and 2) those who had not received antidiabetic drugs for two or more times at outpatient follow-up (Tseng, 2015a).

We then excluded the following ineligible patients in the second step exclusion: 1) women; 2) patients with type 1 diabetes mellitus; 3) patients with missing data; 4) patients aged <18 years at the start of follow-up; 5) patients aged >80 years at the start of follow-up; 6) patients with a diagnosis of any cancer before the start of follow-up; 7) patients with any diagnosis of diseases of male genital organs before the start of follow-up; 8) patients with any diagnosis of the investigated outcomes; and 9) patients with available data of exposure assessment for <12 months.

### 2.5 Potential confounders

The following categories of variables were considered as potential confounders: I basic data, II comorbidities commonly associated with diabetes mellitus, III diabetes complications, IV comorbidities that might affect antidiabetic prescription or the investigated outcomes, V medications commonly used in patients with diabetes mellitus, and VI antidiabetic drugs.

The time elapsed since diabetes diagnosis until the first prescription of antidiabetic drugs was defined as “time since diagnosis of diabetes.” The living regions of the patients were classified into the following five categories according to the locations of the branch offices of the Bureau of NHI in different geographical regions (Tseng, 2019): Taipei, Northern, Central, Southern, and Kao-Ping/Eastern. Occupation was divided into four classes according to the Bureau of NHI (Tseng, 2019): (I) civil servants, teachers, employees of governmental or private businesses, professionals and technicians; (II) people without a specific employer, self-employed people and seamen; (III) farmers and fishermen; and (IV) low-income families supported by social welfare and veterans.

Medications commonly used in patients with diabetes mellitus or medications that may affect the investigated outcomes included in the analyses were: angiotensin converting enzyme inhibitor/angiotensin receptor blocker, calcium channel blocker, statin, fibrate, aspirin, alpha blocker, and immunosuppressant. Immunosuppressant was defined by a consistent use of corticosteroids, calcineurin inhibitors and/or inosine-5′-monophosphate dehydrogenase inhibitors for a period of 90 days or more. Antidiabetic drugs included insulin, sulfonylurea, meglitinide, acarbose, rosiglitazone and pioglitazone. At the start of follow-up, metformin was prescribed to all patients in the metformin (+) group and to none of the patients in the metformin (−) group.

### 2.6 Statistical analyses

Statistical analyses were conducted by using version 9.4 of SAS statistical software (SAS Institute, Cary, NC, United States). *p* < 0.05 was considered as having statistical significance.

Standardized difference was used to evaluate the balance of characteristics between metformin (+) and metformin (−) according to Austin and Stuart (Austin and Stuart, 2015). Potential confounding was considered when a value of standardized difference was >10%.

A clinical trial always compares the risk of an investigated outcome between patients randomized to a target drug and those to a control drug or placebo, using both approaches of intention-to-treat (ITT) and per-protocol (PP) analyses. In order to emulate a target trial, we analyzed the data comparing the risk of the investigated outcomes in metformin (+) to metformin (−), taking both the ITT and the PP approaches. In ITT analysis, the numerator of the incidence was the number of patients who had a new diagnosis of the investigated outcomes during follow-up. The denominator was calculated as person-years of follow-up, which started after an initial period of 12 months used for the assessment of metformin initiation, and ended when a diagnosis of the outcomes was made, when the patients died or when the last record of reimbursement was available in the database, whichever occurred first up to the date of 31 December 2011. No cases were excluded in the ITT analyses after the start of follow-up because of switching to or adding other antidiabetic drugs.

For PP analyses, we first excluded patients who were not adherent to the treatment assigned during the first 12 months of exposure assessment. The other patients were then followed up, starting at the end of the first 12 months and ended when a diagnosis of the outcomes was made, when the patients died, when the last record of reimbursement was available, or when the treatment assignment was not adhered to, whichever occurred first up to the date of 31 December 2011.

Propensity scores (PS) were estimated by logistic regression with independent variables that included the starting date of follow-up and the characteristics listed in Table 1. Some unmeasured factors (e.g., the changes of treatment guidelines, the improvement of therapeutic modalities, the prolongation of life expectancy and the introduction of newer classes of antidiabetic drugs) evolving during the long inclusion period might have been partially adjusted for by including the follow-up starting date in the estimation of PS. Both ITT and PP hazard ratios comparing metformin (+) to metformin (−) for outcomes of varicocele, ED, infertility, prostatitis, BPH and prostate cancer, respectively, were estimated by Cox regression incorporated with the inverse probability of treatment-weighting (IPTW) using PS. Austin recommended this regression method to reduce confounding resulting from the differences in characteristics between compared groups (Austin, 2013).

**TABLE 1:** Characteristics in non-metformin initiators and metformin initiators.


Variable	Metformin (−)		Metformin (+)		Standardized difference
	(*n* = 86667)		(*n* = 175171)		
	n	%	n	%	
I. Basic data					
Age* (years)	53.57	11.48	51.80	11.30	−17.69
Time since diabetes diagnosis* (years)	1.46	1.20	1.66	1.32	19.76
Occupation					
I	37571	43.35	80726	46.08	
II	17468	20.16	36229	20.68	1.95
III	16902	19.50	28648	16.35	−8.92
IV	14726	16.99	29568	16.88	−0.70
Living region					
Taipei	26829	30.96	60803	34.71	
Northern	9572	11.04	22390	12.78	5.59
Central	14959	17.26	31043	17.72	2.14
Southern	15625	18.03	26988	15.41	−7.52
Kao-Ping and Eastern	19682	22.71	33947	19.38	−9.18
II. Comorbidities commonly associated with diabetes					
Hypertension	45277	52.24	95023	54.25	4.53
Dyslipidemia	37601	43.39	94684	54.05	23.05
Obesity	1097	1.27	5253	3.00	11.53
III. Diabetes complications					
Nephropathy	9401	10.85	17424	9.95	−1.92
Eye diseases	3421	3.95	12172	6.95	13.91
Diabetic polyneuropathy	4713	5.44	13831	7.90	11.44
Stroke	11397	13.15	22054	12.59	−1.53
Ischemic heart disease	19074	22.01	37787	21.57	−1.03
Peripheral arterial disease	6868	7.92	15279	8.72	3.89
Hypoglycemia	443	0.51	872	0.50	0.42
IV. Comorbidities that might affect antidiabetic prescription or outcomes					
Chronic obstructive pulmonary disease	22808	26.32	47769	27.27	2.32
Tobacco abuse	1291	1.49	4538	2.59	7.98
Alcohol-related diagnoses	5825	6.72	12314	7.03	2.28
Heart failure	6113	7.05	10833	6.18	−3.49
Parkinson’s disease	648	0.75	1169	0.67	−1.06
Dementia	2105	2.43	3590	2.05	−2.94
Head injury	520	0.60	520	0.30	3.87
Valvular heart disease	3305	3.81	6004	3.43	−2.46
Helicobacter Pylori infection	11510	13.28	24799	14.16	3.12
Epstein-Barr virus infection	306	0.35	682	0.39	0.81
Hepatitis B virus infection	956	1.10	3000	1.71	5.54
Hepatitis C virus infection	1817	2.10	4055	2.31	2.14
Human immunodeficiency virus disease	57	0.07	111	0.06	0.12
Cirrhosis of liver without mention of alcohol	3250	3.75	5356	3.06	−3.04
Other chronic nonalcoholic liver disease	4777	5.51	11774	6.72	5.40
Autoimmune diseases	3045	3.51	6638	3.79	1.69
Organ transplantation	174	0.20	191	0.11	−2.14
Insomnia	8171	9.43	18925	10.80	5.43
Malaise and fatigue	1582	1.83	5235	2.99	8.01
Episodic mood disorders	1694	1.95	4015	2.29	2.52
Syphilis and other venereal diseases	668	0.77	1700	0.97	2.30
History of some disorders of the central nervous system	9571	11.04	20026	11.43	1.49
Benign neoplasm of bone and articular cartilage	144	0.17	369	0.21	1.00
Osteoporosis	3669	4.23	7591	4.33	0.76
Any bone fractures	10596	12.23	24631	14.06	6.00
Ocular pterygium	1539	1.78	3333	1.90	1.19
Disorders of thyroid gland	2502	2.89	6721	3.84	5.64
Nutritional deficiencies	1175	1.36	2162	1.23	−0.98
Urinary tract infection	7445	8.59	17063	9.74	4.77
Retention of urine	79	0.09	191	0.11	0.59
Urinary obstruction	141	0.16	339	0.19	0.80
Calculus of kidney and ureter	10283	11.86	24238	13.84	6.45
Calculus of lower urinary tract	984	1.14	2365	1.35	2.10
V. Medications that are commonly used or may affect disease risk					
Angiotensin converting enzyme inhibitor/angiotensin receptor blocker	34146	39.40	73736	42.09	5.84
Calcium channel blocker	32096	37.03	60597	34.59	−5.60
Statin	19163	22.11	51460	29.38	17.45
Fibrate	19088	22.02	44339	25.31	8.14
Aspirin	26956	31.10	57171	32.64	3.33
Alpha blocker	8156	9.41	8156	4.66	−2.99
Immunosuppressant	2302	2.66	3963	2.26	−2.53
VI. Antidiabetic drugs					
Insulin	6051	6.98	14461	8.26	9.17
Sulfonylurea	77771	89.74	129023	73.66	−40.01
Meglitinide	4482	5.17	10523	6.01	7.43
Acarbose	5498	6.34	11287	6.44	4.78
Rosiglitazone	2217	2.56	7286	4.16	10.24
Pioglitazone	1384	1.60	4810	2.75	9.03

In order to examine the consistency of the findings, we performed the following sensitivity analyses for both ITT and PP analyses. First, varicocele diagnosis was replaced by a more stringent definition of varicocelectomy (ICD-9-CM operation code: 63.1). Second, separate analyses were conducted for outcomes of organic ED (607.84), psychogenic ED (302.72) and ED diagnosed by urologists. Third, analyses were conducted for an outcome of receiving surgical procedure for prostate (operation codes: 60.2, 60.3, 60.4, 60.5, and 60.6).

## 3 Results

**FIGURE 1:**
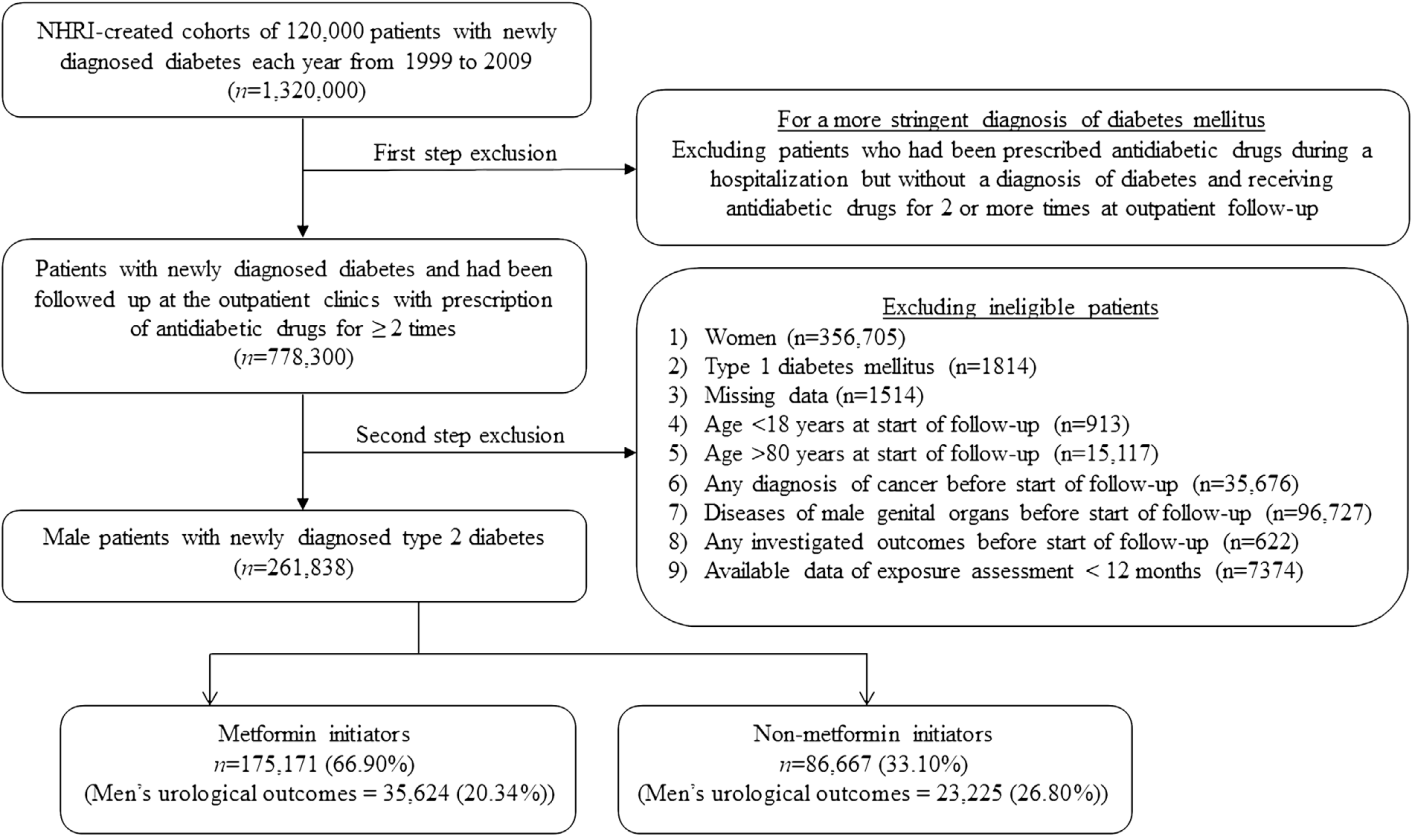
Flowchart showing the procedures used in the creation of a cohort of metformin initiators and non-metformin initiators from Taiwan’s National Health Insurance database. Investigated men’s urological outcomes include any of the following: varicocele, erectile dysfunction, infertility, prostatitis, benign prostate hyperplasia, and prostate cancer. NHRI, National Health Research Institutes.

Figure 1 shows the procedures followed in the creation of the study subjects derived from the nationwide database of the NHI. After the first step exclusion, we identified 778,300 patients who had a diagnosis of diabetes mellitus between 1999 and 2009 in the outpatient clinics and had been prescribed antidiabetic drugs for ≥2 times. After the second step exclusion of ineligible patients, 261,838 male patients with T2DM newly diagnosed during 1999–2009 were enrolled into the study. Among them, 175,171 were metformin (+) and 86,667 were metformin (−).

Characteristics in metformin (+) and metformin (−) are shown in Table 1. Standardized difference >10% was observed in the following variables: age, time since diagnosis of diabetes, dyslipidemia, obesity, eye diseases, diabetic polyneuropathy, statin, sulfonylurea, and rosiglitazone, indicating potential confounding from these variables.

Table 2 shows the incidence rates of the respective outcomes in metformin (+) and metformin (−) and the estimated hazard ratios comparing metformin (+) to metformin (−) in both the ITT analyses and the PP analyses. The results in both the ITT and the PP analyses were consistent and suggested that metformin use was associated with a neutral effect of varicocele, a significantly higher risk of ED and male infertility, and a significantly lower risk of prostate-related outcomes including prostatitis, BPH and prostate cancer.

**TABLE 2:** Incidence rates of outcomes and hazard ratios comparing metformin initiators to non-metformin initiators.

Outcome/Metformin use	Incident case number	Cases followed	Median follow-up time (years)	Person-year	Incidence rate (per 100,000 person-years)	Hazard ratio	95% confidence interval	*p* value
Varicocele								
Intention-to-treat								
Metformin (−)	154	86667	6.82	582919.74	26.42	1.000		
Metformin (+)	248	175171	5.17	966690.64	25.65	0.960	(0.784–1.174)	0.6904
Per-protocol								
Metformin (−)	96	86667	2.61	307250.23	31.24	1.000		
Metformin (+)	206	153352	4.65	780049.79	26.41	0.845	(0.662–1.078)	0.1753
Erectile dysfunction								
Intention-to-treat								
Metformin (−)	2608	86667	6.65	572065.76	455.89	1.000		
Metformin (+)	4634	175171	5.05	949401.23	488.10	1.077	(1.026–1.130)	0.0026
Per-protocol								
Metformin (−)	1163	86667	2.56	301349.07	385.93	1.000		
Metformin (+)	3981	153352	4.54	765892.04	519.79	1.350	(1.264–1.441)	<0.0001
Male infertility								
Intention-to-treat								
Metformin (−)	133	86667	6.82	582946.53	22.82	1.000		
Metformin (+)	315	175171	5.17	966357.48	32.60	1.368	(1.116–1.676)	0.0025
Per-protocol								
Metformin (−)	75	86667	2.61	307294.61	24.41	1.000		
Metformin (+)	252	153352	4.65	779826.74	32.31	1.396	(1.078–1.808)	0.0115
Prostatitis								
Intention-to-treat								
Metformin (−)	3366	86667	6.63	570290.74	590.23	1.000		
Metformin (+)	4850	175171	5.05	950421.47	510.30	0.887	(0.849–0.927)	<0.0001
Per-protocol								
Metformin (−)	1817	86667	2.55	299402.73	606.87	1.000		
Metformin (+)	3868	153352	4.55	767690.85	503.85	0.800	(0.756–0.846)	<0.0001
Benign prostate hyperplasia								
Intention-to-treat								
Metformin (−)	21022	86667	5.55	497422.36	4226.19	1.000		
Metformin (+)	31478	175171	4.36	854067.36	3685.66	0.883	(0.868–0.899)	<0.0001
Per-protocol								
Metformin (−)	10346	86667	2.20	256836.04	4028.25	1.000		
Metformin (+)	25023	153352	3.99	696213.46	3594.16	0.875	(0.855–0.895)	<0.0001
Prostate cancer								
Intention-to-treat								
Metformin (−)	823	86667	6.78	580830.50	141.69	1.000		
Metformin (+)	1124	175171	5.16	964217.24	116.57	0.878	(0.802–0.961)	0.0049
Per-protocol								
Metformin (−)	495	86667	2.60	305837.14	161.85	1.000		
Metformin (+)	825	153352	4.64	778294.29	106.00	0.613	(0.548–0.686)	<0.0001

The findings of the main analyses in Table 2 were also supported by sensitivity analyses shown in Table 3, indicating a neutral effect of varicocelectomy, a higher risk of ED by different definitions and a reduced risk of receiving surgical procedures for prostate among the metformin (+) group.

**TABLE 3:** Sensitivity analyses.

Outcome/Metformin use	Incident case number	Cases followed	Median follow-up time (years)	Person-year	Incidence rate (per 100,000 person-years)	Hazard ratio	95% confidence interval	*p* value
Varicocelectomy								
Intention-to-treat								
Metformin (−)	48	86666	6.82	583380.29	8.23	1.000		
Metformin (+)	96	175170	5.18	967247.80	9.93	1.185	(0.838–1.677)	0.3376
Per-protocol								
Metformin (−)	28	86666	2.61	307596.81	9.10	1.000		
Metformin (+)	74	153351	4.66	780493.19	9.48	1.024	(0.662–1.583)	0.9156
Organic erectile dysfunction								
Intention-to-treat								
Metformin (−)	2439	86667	6.67	572977.13	425.67	1.000		
Metformin (+)	4288	175171	5.06	950837.37	450.97	1.068	(1.016–1.122)	0.0103
Per-protocol								
Metformin (−)	1082	86667	2.57	301877.46	358.42	1.000		
Metformin (+)	3680	153352	4.55	767099.74	479.73	1.338	(1.250–1.433)	<0.0001
Psychogenic erectile dysfunction								
Intention-to-treat								
Metformin (−)	275	86667	6.80	582167.69	47.24	1.000		
Metformin (+)	527	175171	5.16	965422.19	54.59	1.155	(0.998–1.338)	0.0531
Per-protocol								
Metformin (−)	136	86667	2.61	306873.81	44.32	1.000		
Metformin (+)	464	153352	4.64	778883.21	59.57	1.358	(1.121–1.645)	0.0018
Erectile dysfunction diagnosed by urologists								
Intention-to-treat								
Metformin (−)	2280	86667	6.68	573617.31	397.48	1.000		
Metformin (+)	4079	175171	5.07	951713.26	428.60	1.088	(1.033–1.145)	0.0013
Per-protocol								
Metformin (−)	1011	86667	2.57	302219.16	334.53	1.000		
Metformin (+)	3503	153352	4.56	767786.73	456.25	1.362	(1.270–1.462)	<0.0001
Surgical procedures for prostate								
Intention-to-treat								
Metformin (−)	1128	86666	6.76	579075.73	194.79	1.000		
Metformin (+)	1392	175170	5.15	962964.91	144.55	0.786	(0.727–0.851)	<0.0001
Per-protocol								
Metformin (−)	693	86666	2.59	304572.27	227.53	1.000		
Metformin (+)	1021	153351	4.64	777531.63	131.31	0.535	(0.486–0.590)	<0.0001

A graphical abstract illustrating the follow-up timeline axis and the main findings of the study is presented as a Supplementary Material.

## 4 Discussion

### 4.1 Main findings

This study first investigated simultaneously the risk of various urological outcomes of men’s health including varicocele, ED, male infertility, prostatitis, BPH, and prostate cancer associated with metformin use. The findings suggesting different effects of metformin on different outcomes are interesting and have not been reported previously. Metformin seemed to have a neutral effect on varicocele. Although a significantly reduced risk of prostate diseases including prostatitis, BPH and prostate cancer was observed in association with metformin use, metformin might on the other hand adversely affect sexual function of ED and infertility (Tables 2, 3).

Because metformin would exert different effects on different men’s urological outcomes, a composite outcome that includes all the investigated outcomes of the study should not be used in future research. In secondary analyses, when we included varicocele, ED, infertility, prostatitis, BPH, and prostate cancer as a composite outcome, the hazard ratio that compared metformin (+) to metformin (−) was 0.900 (0.886–0.916) in the ITT analysis and was 0.916 (0.896–0.963) in the PP analyses. The results would suggest a preventive role of metformin on the composite outcome.

### 4.2 Metformin has a neutral effect on varicocele

Varicocele is a disease characterized by venous dilatation involving the internal spermatic veins (Paick and Choi, 2019). The neutral effect of metformin on varicocele defined by a clinical diagnosis (Table 2) or by varicocelectomy (Table 3) was contradictory to a reduced risk of another venous disease of varicose veins associated with metformin use observed in our previous study (Tseng, 2020). The reasons for such discrepant findings remain to be explored, but low incidence and under-diagnosis of varicocele are possible explanations for the lack of an association. It has been reported that approximately 15% of men may have varicocele but only 2%–10% of them would complain of scrotal pain, the most common clinical presentation (Paick and Choi, 2019). Furthermore, Asian patients with diseases of the sexual organs would also be reluctant to consult their clinical doctors because of embarrassment. Therefore, the lower rates of clinical symptoms and doctor consultations might have led to under-diagnosis of varicocele. Together with the lower incident case numbers, a lack of statistical power was possible. Another possible reason is the misclassification of disease diagnosis by using the disease diagnostic code because nondifferential misdiagnosis would have led to an estimated risk biased toward the null (Kesmodel, 2018). However, because misclassification by using a more stringent operation code of “varicocelectomy” (Table 3) was less likely, the consistent findings observed in the sensitivity analyses did support a neutral effect of metformin on varicocele. It is worthy to note that patients who received varicocelectomy might only represent those with more severe clinical presentations that required a surgical intervention. Although a previous Taiwanese study suggested that varicocele and varicose veins may have a high association (Lai et al., 2015), another study conducted in Turkey that compared various venous parameters between varicocele and varicose veins by using color Doppler ultrasonography showed a lack of statistically significant relation and concluded that “varicocele may not be attributable to a systemic vascular insufficiency” (Yazici et al., 2012). Therefore, the benefit of metformin on varicose veins (Tseng, 2020) should not be extrapolated to varicocele.

### 4.3 Metformin may adversely affect sexual functions

Several animal studies suggested a potential benefit of metformin on ED (Silva et al., 2015; Zhang et al., 2020). This was also shown by an early pilot trial in humans with small sample size (Rey-Valzacchi et al., 2012). This small clinical trial investigated the sexual function after addition of metformin to patients who had ED but responded poorly to sildenafil in 30 (17 were assigned metformin) men who were not diabetic but had insulin resistance (Rey-Valzacchi et al., 2012). However, this benefit could not be similarly demonstrated in two later human studies (Al-Kuraishy and Al-Gareeb, 2016; Abdul-Hadi et al., 2020). These later human observational studies suggested on the contrary that diabetes patients who used metformin had a lower testosterone level, a reduced sex drive and a higher rate of low testosterone-induced ED (Al-Kuraishy and Al-Gareeb, 2016; Abdul-Hadi et al., 2020). It is interesting that the clinical trial that showed a benefit of metformin on ED recruited men without diabetes but had insulin resistance (Rey-Valzacchi et al., 2012); on the contrary, the later studies suggesting a potential adverse effect were conducted in the diabetes patients (Al-Kuraishy and Al-Gareeb, 2016; Abdul-Hadi et al., 2020). Because psychosocial problems, clinical comorbidities and medications used in various clinical conditions may also affect ED, the lack of consideration of these potential confounders in these previous studies should be pointed out. Furthermore, metformin may exert different effects on ED between diabetes patients and non-diabetes men.

Infertility can be related to testicular functions including testosterone steroidogenesis and spermatogenesis (Tseng, 2022). In diabetic and non-diabetic rabbits, metformin treatment may significantly reduce testicular weight and serum testosterone level together with reduced spermatic count, motility and viability (Naglaa et al., 2010). Another study conducted in male fetal NMRI mice suggested that metformin exposure *in utero* may cause increased lactate production, leading to decreased testosterone secretion, smaller size of testicles and reduced number of Sertoli cells (Tartarin et al., 2012). However, contradictory findings have been noted by other investigators who showed that metformin administration to male Sprague-Dawley rats might protect against testicular damages (Nna et al., 2020). Others showed a restoration of steroidogenesis and spermatogenesis after metformin administration to male Wistar rats (Derkach et al., 2020). In humans, contradictory and controversial findings have also been reported. Tertti et al. (2016) reported that pre-pubertal testicular sizes did not differ significantly between boys who were born to mothers with gestational diabetes mellitus having been treated with metformin and mothers having been treated with insulin during pregnancy. An Italian study suggested that treatment of metformin to obese individuals with metabolic syndrome for 6 months might increase serum testosterone level (Morgante et al., 2011). However, another group from Iraq reported that men with T2DM treated with metformin had a lower testosterone level, a reduced sex drive and a higher rate of low testosterone-induced ED (Al-Kuraishy and Al-Gareeb, 2016; Abdul-Hadi et al., 2020).

Recent studies suggested that maternal and paternal exercise may affect the metabolic health in adult offspring (Zheng et al., 2020) and sperm epigenome can be epigenetically modified by environmental factors and the health of the offspring can be affected by the transgenerational passage of the modified epigenome (Xu et al., 2021). Metformin may regulate epigenetic modifications (Menendez, 2020) and these have been considered as one of the mechanisms explaining the beneficial effects of metformin on the prevention of cancer (Cuyàs et al., 2021) and aging (Menendez, 2020). Some recent studies suggested that *in utero* exposure to metformin may reduce fertility in male offspring in adulthood (Faure et al., 2021) and that maternal exposure to metformin may induce epigenetic modifications via placental mitochondrial biogenesis (Jiang et al., 2020). Whether the effects of metformin on ED and male infertility can be derived from parents or passed to offspring have not been researched. It is worthy to carry out more experimental research and human studies to explore the possibility of parental transmission of epigenetic modifications on steroidogenesis and spermatogenesis induced by metformin to the offspring.

Although we did not have biomarkers of steroidogenesis and spermatogenesis for more detailed analyses, the findings from this large administrative database did suggest for the first time that patients with T2DM being treated with metformin might have an increased risk of ED and infertility (Tables 2, 3). These potential adverse effects of metformin should better be more intensively studied and confirmed or refuted for more appropriate prescription of metformin to patients with related clinical problems.

It is true that diabetes per se and the severity of diabetes and its related complications, comorbidities and use of medications may affect the risk of ED. However, we believe that our findings here should be robust because we have addressed the potential risk of confounding by indication by using the IPTW method and have especially considered additional analyses by using three different definitions of ED: “organic ED,” “psychogenic ED,” and “ED diagnosed by urologists” (Table 3). All analyses were very consistent and suggested a higher risk associated with metformin use.

### 4.4 Metformin may reduce prostate diseases

Metformin seemed to exert beneficial effects on the prostate gland in all aspects of clinical diseases including prostatitis, BPH, and prostate cancer (Tables 2, 3). The effect of metformin on prostatitis has not been previously researched. However, metformin does exert antimicrobial effects against bacteria, viruses, and fungi (Tseng, 2018a; Tseng, 2018b; Nath et al., 2020) and novel metformin-containing compounds are under development for antimicrobial use (Chen et al., 2018a). Therefore, metformin may reduce prostatitis by improving glycemic control and through its actions of anti-inflammation, anti-microorganism and immune modulation.

The benefits of metformin on BPH can be demonstrated in cellular and animal studies and it can act through the regulation of cell cycle or through a reduced expression of insulin-like growth factor 1 and its related receptor (Tseng, 2022). In humans, a Korean study suggested a reduced risk of BPH in metformin users (Hong et al., 2019), though another United States study showed a lack of association (Murff et al., 2014). The present study supported the findings of the Korean study of a potential benefit of metformin in reducing the risk of BPH (Hong et al., 2019).

With regards to prostate cancer, many cellular and animal studies showed that metformin may inhibit the proliferation, migration and progression of cancer cells (Tseng, 2022). The mechanisms may involve a wide range of cellular actions of metformin. In prostate cancer cells, metformin activates the 5’ adenosine monophosphate-activated protein kinase, blocks cell cycle in G_0_/G_1_ by reducing cyclin D1, inhibits the signaling of mammalian target of rapamycin complex 1, blocks androgen receptor, inhibits tumor-associated inflammatory infiltration, inhibits the signaling pathway of insulin-like growth factor 1, downregulates *c-myc* oncogene and targets cellular senescence etc. (Tseng, 2022). In humans, as previously observed in studies conducted in Taiwan (Tseng, 2014a) and Denmark (Preston et al., 2014), metformin use was associated with a reduced risk of prostate cancer. The present study also supported such a benefit associated with metformin use (Tables 2, 3). However, this benefit could not be similarly observed by other investigators and meta-analyses did not conclude consistently (Chen et al., 2018b; He et al., 2019; Wang et al., 2020).

### 4.5 Study limitations

Some potential limitations deserve discussion. First, only clinical diagnoses were used for defining the outcomes in the main analyses (Table 2) and no laboratory data were available. Misclassification of disease diagnoses was possible in the database. However, if the misclassification was not differential, it was expected that the estimated hazard ratios were more likely to bias toward the null (Kesmodel, 2018). Because the findings in the sensitivity analyses were consistent by using more stringent definitions (Table 3), the results should be robust with regards to the different outcomes investigated.

Second, it is recognized that disease severity, comorbidities, contraindications and the use of other medications etc. may affect the physicians’ choice among medications available for the treatment of diabetes. Therefore, the baseline characteristics of the patients who are prescribed a certain medication may not be the same as those who are not. This imbalance in baseline characteristics may lead to a wrong conclusion known as “confounding by indication.” The imbalanced distributions in some covariates were also noted between metformin (+) and metformin (−) in the present study (Table 1). Austin compared and discussed four methods by using the PS to address such a potential risk of confounding by indication and concluded that the Cox regression incorporated with the IPTW method is the best option (Austin, 2013). In secondary analyses, we also analyzed the data by using a matched cohort of metformin (+) and metformin (−) based on PS and by covariate adjustment using PS. The findings were very similar and the conclusions were not affected (data not shown). Therefore, the results and conclusions would not be affected by the use of different methods.

Third, insulin injection is always the last resort for the treatment of T2DM in Taiwan and therefore, its use is always associated with a prolonged duration of diabetes and a more severe disease condition characterized by more diabetes complications and more complicated use of other medications. It is true that insulin use has been found to be associated with an increased risk of hypertension (Tseng, 2006), obesity (Tseng, 2007), peripheral arterial disease (Tseng, 2003) and several types of cancer (Hsieh et al., 2012; Tseng, 2013a; Tseng, 2015b; Tseng, 2015c) in our previous studies. The small proportion of insulin use in our patients as shown in Table 1, 8.26% among metformin (+) and 6.98% among metformin (−), was consistent with our early study that used a questionnaire to interview patients with T2DM in mid-1990s that showed an insulin using rate of 6.7% among 87,850 patients (Tseng, 2006). The slightly higher proportion of insulin use among metformin (+) might not have affected the conclusion of our study because the standardized difference of 9.17% (Table 1) did not meet the cutoff threshold of >10% to indicate potential confounding. Additionally, its impact might have been weighted by the IPTW method applied in the Cox regression. Because insulin use was not associated with prostate cancer risk in terms of incidence (Tseng, 2014b) or mortality (Tseng, 2012) in our previous studies, its use might not have played a confounding role in the analyses for prostate cancer.

Fourth, although we have tried to address confounding by indication by using the IPTW method, we still recognized that unmeasured confounders could never be adjusted for by statistical methods. Therefore, in the assessment of a cause-effect relationship, retrospective observational studies cannot be as good as randomized clinical trials in which unmeasured confounders are supposed to be evenly distributed in both the treatment group and the control group through randomization. However, if unmeasured confounders are associated with measured confounders, adjustment for measured confounders may also have adjusted for the unmeasured ones (Stuart, 2010; Kuss et al., 2016).

Finally, it was noted that in the ITT analyses, the metformin (−) group had a longer follow-up time than the metformin (+) group. However, in the PP analyses, the metformin (−) group had a shorter follow-up time than the metformin (+) group. There are two possible explanations for such a discrepancy of follow-up times in ITT and PP analyses. First, patients in the metformin (−) group were more likely to be added on metformin because metformin had been recommended as the first-line treatment after the start of follow-up. On the other hand, patients in the metformin (+) group would be more likely to be continuously treated with metformin after follow-up because of its multiple benefits found in recent years. Despite the discrepant follow-up times in the ITT and PP analyses, the results derived from either analysis were consistent and the ITT and PP hazard ratios were in the same direction for each specific outcome investigated (Tables 2, 3, Supplementary Material). The consistent findings by using different approaches strongly supported the robustness of the findings.

### 4.6 Strengths of the study

This study has some merits that deserve mentioning. First, there has not been any previous study that evaluated multiple outcomes related to men’s urological health simultaneously in a single study.

Second, the competing risk of death has been addressed in the person-years calculation of follow-up duration.

Third, because the NHI is a unique and universal healthcare system in Taiwan and the present study recruited a large sample size of diabetes patients randomly selected within a national scale, the risk of selection bias should be very small and therefore the findings could be immediately applied to the whole population.

Fourth, the temporal correctness of cause (metformin exposure) and effects (the investigated outcomes) could be assured by using the NHI database that contained longitudinal information.

Fifth, prevalent user bias and immortal time bias associated with the use of administrative databases in the real world have been carefully addressed. In order to avoid prevalent user bias, only patients who had a new diagnosis of T2DM were included and we started follow-up at a time after the first prescription of antidiabetic drugs. Immortal time is the duration included in the calculation of follow-up person-years during which the outcome cannot occur. Inappropriate assignment of treatment status or inappropriate assignment of follow-up time can lead to immortal time bias. Misdiagnosis of diabetes mellitus was very unlikely because, in addition to a diagnosis of diabetes mellitus, patients should also had been prescribed antidiabetic drugs for at least 2 times (Figure 1). Misclassification of treatment status was also unlikely because all longitudinal information of drug prescriptions during the long follow-up duration was available in the nationwide NHI database. The immortal time that could happen during the period between the diagnosis of diabetes and the first prescription of antidiabetic drugs (“time since diagnosis of diabetes”) had been addressed in the logistic regression that estimated the PS (Table 1) and this time period was not considered in the calculation of follow-up time to avoid a potential risk of immortal time bias. In the NHI healthcare system in Taiwan all drugs prescribed at hospital discharge can be immediately refilled at the hospital. Therefore, the immortal time that could be introduced during the waiting time between hospital discharge and the refill of the drugs would not happen here.

Sixth, the use of preexisting medical records prevented recall bias and other biases related to self-reporting.

Seventh, detection bias resulting from varying socioeconomic status might not be a significant issue in the present study because the medical copayments are very cheap. Furthermore, much of the cost can be waived in the NHI system for patients who have low-income, for veterans and for those who receive refills of drugs for chronic diseases.

### 4.7 Clinical implications

The present study may have some clinical implications. First, because the effects of metformin on varicocele, sexual function (i.e., ED and infertility) and prostate-related health (prostatitis, BPH, and prostate cancer) were not the same, it would be more appropriate to detail the use of metformin with regards to the different clinical conditions of the patients.

Second, the consistent findings of a reduced risk of prostate-related outcomes including prostatitis, BPH and prostate cancer (Table 2) and a reduced incidence of surgical procedures for prostate performed among metformin (+) (Table 3) provided evidence for metformin use for glycemic control in older male patients if they do not have a high demand of sexual activity and fertility because metformin may provide an extra bonus of reducing the risk of age-related prostate diseases. The larger magnitude of risk reduction of prostate-related outcomes (Table 2) and surgical procedures for prostate (Table 3) in PP analyses in comparison to ITT analyses implied a better protective effect of metformin if the patients could adhere to the use of metformin.

Third, BPH may increase the risk of bladder cancer in patients with T2DM in Taiwan (Tseng, 2013b) and metformin use may reduce the risk of several types of cancer including bladder cancer (Tseng, 2014c). Therefore, the reduced risk of BPH associated with metformin may also contribute to a reduced risk of bladder cancer associated with its use. Because prostate cancer incidence (Tseng, 2011a) and mortality (Tseng, 2011b) are on the rise in Taiwan and metformin has potential benefits on the reduction of many other types of cancer (Tseng, 2014d; Tseng, 2014e; Tseng, 2016; Tseng, 2021), it is rationale to recommend metformin as the first-line therapy for patients with T2DM, especially in those with a high risk of cancer.

Fourth, the higher magnitude of hazard ratios for ED (Tables 2, 3) and male infertility (Table 2) in PP analyses than in ITT analyses might have implied a cause-effect relationship with regards to the adherence to treatment. These potential adverse effects of metformin should be attended and confirmation with more in-depth investigation and in other ethnicities is needed. At least for the moment metformin should better be avoided in younger male patients who have a demand for fecundity or a more satisfactory sexual life in the Taiwanese patients with T2DM.

## 5 Conclusion

This observational study is the first to simultaneously investigate metformin’s multiple effects on men’s urological health with regards to varicocele, ED, infertility, prostatitis, BPH, and prostate cancer. The findings suggest a lack of association with varicocele, a significantly higher risk of sexual dysfunctions of ED and infertility, and a significant risk reduction of prostate diseases including prostatitis, BPH, and prostate cancer.

## Data Availability

The original contributions presented in the study are included in the article/Supplementary Materials, further inquiries can be directed to the corresponding author.

## Abbreviations

BPH, benign prostate hyperplasia; ED, erectile dysfunction; ICD-9-CM, international classification of diseases, ninth revision, clinical modification; IPTW, inverse probability of treatment weighting; ITT, intention-to-treat; Metformin (+), metformin initiators; Metformin (−), non-metformin initiators; NHI, national health insurance; NHRI, national health research institutes; PP, per-protocol; PS, propensity scores; T2DM, type 2 diabetes mellitus.
