# Supplementary material for: Metformin’s effects on varicocele, erectile dysfunction, infertility and prostate-related diseases: A retrospective cohort study
Source: Front Pharmacol. 2022 Jul 22;13:799290. doi: 10.3389/fphar.2022.799290 (PMC9355151; doi:10.3389/fphar.2022.799290)
Supplement: Supplementary file 1 [file Table1.docx]

Supplementary Table S1. Disease diagnoses used in the study and their corresponding codes according to the International Classification of Diseases, Ninth Revision, Clinical Modification (ICD-9-CM)

| Disease diagnoses | ICD-9-CM codes |
| --- | --- |
| Diabetes mellitus | 250.XX |
| Diseases of male genital organs | 600–608 |
| Varicocelectomy | 63.1 (operation code) |
| Surgical procedure for prostate | 60.2, 60.3, 60.4, 60.5 and 60.6 (operation codes) |
| Varicocele | 456.4 |
| Erectile dysfunction | 302.72: psychogenic erectile dysfunction  607.84: organic erectile dysfunction |
| Male infertility | 606 |
| Prostatitis | 601 |
| Benign prostate hyperplasia | 600 |
| Prostate cancer | 185 |
| Any cancer | 140–208 |
| Hypertension | 401–405 |
| Dyslipidemia | 272.0–272.4 |
| Obesity | 278 |
| Nephropathy | 580–589 |
| Eye diseases | 250.5: diabetes with ophthalmic manifestations  362.0: diabetic retinopathy  369: blindness and low vision  366.41: diabetic cataract  365.44: glaucoma associated with systemic syndromes |
| Diabetic polyneuropathy | 357.2, 250.6 |
| Stroke | 430–438 |
| Ischemic heart disease | 410–414 |
| Peripheral arterial disease | 250.7, 785.4, 443.81, 440–448 |
| Hypoglycemia | 251.0, 251.1, 251.2 |
| Chronic obstructive pulmonary disease | 490–496 |
| Tobacco abuse | 305.1, 649.0, 989.84 |
| Alcohol-related diagnoses | 291, 303, 535.3, 571.0–571.3, 980.0 |
| Heart failure | 398.91, 402.11, 402.91, 404.11, 404.13, 404.91, 404.93, 428 |
| Parkinson’s disease | 332 |
| Dementia | 290.0, 290.1, 290.2, 290.4, 294.1, 331.0–331.2, 331.7–331.9 and/or abridged codes of A210 or A222 |
| Head injury | 959.01 |
| Valvular heart disease | 394–396, 424, 746 |
| Helicobacter pylori infection | 041.86 |
| Epstein-Barr virus infection | 075, 710.3, 710.4 |
| Hepatitis B virus infection | 070.22, 070.23, 070.32, 070.33, V02.61 |
| Hepatitis C virus infection | 070.41, 070.44, 070.51, 070.54, V02.62 |
| Human immunodeficiency virus disease | 042 |
| Cirrhosis of liver without mention of alcohol | 571.5 |
| Other chronic nonalcoholic liver disease | 571.8 |
| Autoimmune diseases | 579.0: celiac disease  696.0: psoriatic arthritis  696.1: psoriasis  710.0: systemic lupus erythematosus  710.1: systemic sclerosis  710.2: Sjogren’s syndrome  714.0: rheumatoid arthritis |
| Organ transplantation | V42 |
| Insomnia | 780.52 |
| Malaise and fatigue | 780.79 |
| Episodic mood disorders | 296 |
| Syphilis and other venereal diseases | 090–099 |
| History of some disorders of the central nervous system | 340–349 |
| Benign neoplasm of bone and articular cartilage | 213.0–213.9 |
| Osteoporosis | 733.00, 733.01, 733.02, 733.03, 733.09 |
| Any bone fractures | 800–829 |
| Ocular pterygium | 372.40–372.44 |
| Disorders of thyroid gland | 240–246 |
| Nutritional deficiencies | 260–269 |
| Urinary tract infection | 599.0 |
| Retention of urine | 788.20 |
| Urinary obstruction | 599.6 |
| Calculus of kidney and ureter | 592 |
| Calculus of lower urinary tract | 594 |
