# Supplementary material for: Metformin’s effects on varicocele, erectile dysfunction, infertility and prostate-related diseases: A retrospective cohort study
Source: Front Pharmacol. 2022 Jul 22;13:799290. doi: 10.3389/fphar.2022.799290 (PMC9355151; doi:10.3389/fphar.2022.799290)

Supplementary Material:

A simplified snapshot illustrating the follow-up timeline axis and the main findings of the study. NHI: National Health Insurance; NHRI: National Health Research Institutes.


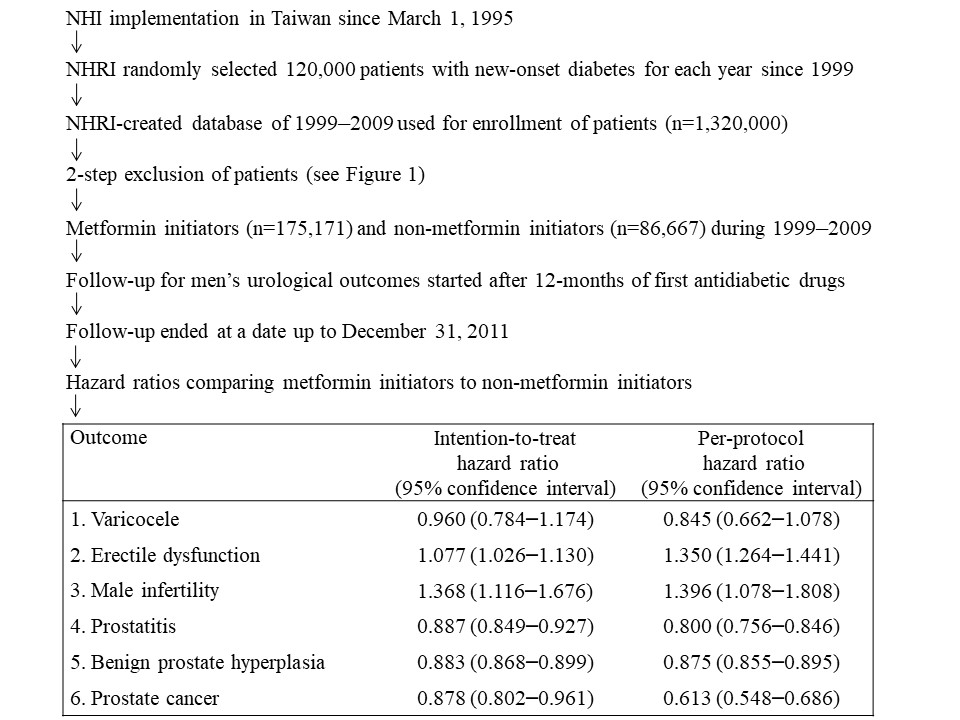

Supplement: Supplementary file 2 [file Table2.docx]
